# Remdesivir inhibits Porcine epidemic diarrhea virus infection in vitro

**DOI:** 10.1016/j.heliyon.2023.e21468

**Published:** 2023-11-02

**Authors:** Zi-Xin Huang, Shu-Ting Zhou, Jing Wang, Zhi-Biao Yang, Zhe Wang

**Affiliations:** a Shanghai Collaborative Innovation Center of Agri-Seeds / School of Agriculture and Biology, Shanghai Jiao Tong University, Shanghai 200240, China; b Shanghai Key Laboratory of Veterinary Biotechnology, School of Agriculture and Biology, Shanghai Jiao Tong University, Shanghai 200240, China; c Department of Preventive Veterinary Medicine, College of Veterinary Medicine, Northwest A&F University, Yang ling, Xianyang 712100, China

**Keywords:** Porcine epidemic diarrhea virus, remdesivir, Replication inhibition, RdRp, RNA-Seq

## Abstract

Porcine Epidemic Diarrhea Virus (PEDV) is a highly contagious and pathogenic virus that causes symptoms such as diarrhea, vomiting, weight loss, and even death in piglets. Due to its high transmission rate, PEDV has resulted in significant global losses. Although some vaccines have been developed and utilized to prevent PEDV, their effectiveness is limited due to the virus's mutations. Therefore, it is imperative to investigate new strategies to combat PEDV. Remdesivir, a classic antiviral drug for coronaviruses, has been proven in our experiment to effectively suppress PEDV replication in Vero and LLC-PK1 cells. Additionally, the cell experiment demonstrated its direct inhibition of PEDV RNA-dependent RNA polymerase (RdRp) enzyme activity. Molecular docking simulations were employed to predict the binding site of remdesivir and PEDV RdRp. Moreover, we observed that remdesivir does not impact the production of inflammatory factors and exhibits antagonistic effects with exogenous nucleosides. Furthermore, we conducted RNA-Seq analysis to investigate the global changes in transcriptome of infected cells treated with remdesivir. Overall, our findings indicate that remdesivir holds promise as a potential candidate for the treatment of PEDV infection.

## Highlights

• Remdesivir inhibits PEDV infection in Vero cells and LLC-PK1 cells in a dose-dependent manner.
• Remdesivir effectively blocks PEDV replication.
• Remdesivir inhibits PEDV RNA-dependent RNA polymerase activity.
• Exogenous Nucleosides Attenuate Antiviral Effect of Remdesivir.
• Remdesivir could recovery the transcriptome changes caused by viral infection.

## 1 Introduction

Porcine epidemic diarrhea virus (PEDV), a member of the Coronaviridae family and Alphacoronavirus genus, was first identified in Belgium in 1978 [1]. It poses a substantial threat to the global pig industry, leading to severe symptoms such as acute diarrhea, vomiting, dehydration, and high mortality rates in newborn piglets. China witnessed a series of widespread PEDV outbreaks in 2010, resulting in significant economic losses [2]. These epidemics were caused by the emergence of highly virulent strains, with mortality rates among neonatal piglets ranging from 50 % to 100 %. Traditional PEDV vaccines, based on classic strains, have proven ineffective in providing adequate protection. Therefore, it is essential to develop alternative methods for preventing and controlling PEDV outbreaks.

PEDV, similar to other coronaviruses, relies on RNA-dependent RNA polymerase (RdRp) for crucial genome transcription and replication processes. Given the relatively conserved structure of RdRp across various viruses [3], it is possible that certain existing nucleoside analogues could exhibit inhibitory effects on PEDV. Remdesivir, a prodrug of GS-441524, serves as a 1-cyano-substituted adenine C-nucleoside ribose analogue. Biochemical studies have shown that RdRp can utilize remdesivir as a substrate within cells, leading to the incorporation of remdesivir monophosphate (RMP) into growing RNA molecules. After the incorporation of RMP, RdRp elongates the RNA strand by three nucleotides, causing transcription to halt [4]. Remdesivir has obtained FDA approval for the treatment of COVID-19 patients, demonstrating its exceptional safety and pharmacokinetic characteristics during the pandemic [5].

Previous studies have demonstrated the inhibitory effects of remdesivir on human coronaviruses, yet its impact on animal coronaviruses remains unclear. Through cell-based assays, we confirmed the inhibitory effect of remdesivir on PEDV RdRp activity and analyzed its impact on the transcriptome of host cells.

## 2 Material and methods

### 2.1 Cells and virus

PEDV can replicate in various pig cell lines, including pig bladder and kidney cells, swine testis cells, intestinal epithelial cells. Besides, African green monkey kidney cells (Vero cells) whose culture medium is added trypsin can support growth of PEDV. We chose Vero cells (ATCC CCL-81) and LLC-PK1 cells as host cell and HEK-293T (ATCC CRL-3216) cells as plasmid expression platform. Vero cells and 293T cells, stored in our laboratory, were both maintained in Dulbecco's modified Eagle's medium (DMEM, Gibco, USA). The culture medium was supplemented with fetal bovine serum (04-001-1A, BIOIND, Israel)at a concentration of 10 %. LLC-PK1 cells, provided by Shanghai Veterinary Research Institute, Chinese Academy of Agricultural Sciences, were maintained in Minimum Essential Medium (MEM, Gibco, USA). All cells were stored in a carbon dioxide cell culture incubator at 37 °C.

The strain of PEDV used was SHpd/2012 (GenBank: **MN508818.1**). For cultivation, the strain was seeded in serum-free DMEM supplemented with 10 μg/mL trypsin.

### 2.2 Compound

Remdesivir (Table 1; CAS ID: 1,809,249-37-3) was purchased from Macklin, China. Dimethyl sulfoxid-e（DMSO）was used to dissolved Remdesivir to get 16.6 mM stock solution. Coelenterazine-h (Table 1; CAS ID: 50909-86-9) was purchased from AAT Bioquest, USA.

**Table 1:** Chemical structures and molecular weights of compounds used in this study.

Lable Name	Chemistry structure	Molecular Weight
Remdesivir（RDV）		MF: C_27_H_35_N_6_O_8_P Mol. Wt. = 602.6 g/mol
Coelenterazine-h		MF: C_26_H_21_N_3_O_2_ Mol.Wt.: 407.5 g/mol

### 2.3 Plasmids

The plasmids which express PEDV nsp 7, nsp 8, and nsp12 were provided by Shanghai Veterinary Research Institute, Chinese Academy of Agricultural Sciences. To generate the reporter plasmid called PEDV-Gluc, we referred the previously published SARS-CoV-2 RdRp activity reporter construct [6]. The PEDV 5′ and 3′ untranslated regions (UTR) were amplified by PCR from Shpd/2012 strain of PEDV. Meanwhile Gaussia luciferase (Gluc) gene was amplified by PCR from pRetroX-tight-Pur-Gluc vector preserved in our Lab. Subsequently, the amplified sequences were connected by overlapping PCR to obtain 5′UTR-Gluc-3′UTR construct. At the same time, the pRetroX-tight-Pur vector was double-cut using *BamH1* and *EcoR1* restriction enzymes. The cut vector was then ligated to the 5′ UTR-Gluc-3′ UTR construct via homologous recombination.

### 2.4 Western blot analysis

In order to obtain lysed cells, we used radioimmunoprecipitation assay (RIPA) lysis buffer, which contained 1 mM phenyl methyl sulfonyl fluoride (PMSF), to lyse cells for 30min at 4 °C on a shaker to ensure complete cell lysis. The BCA Protein Assay Kit (Beyotime, China) was utilized to quantify protein concentrations. Add SDS loading buffer to cell lysates and boil them for 10min. Following this, prepare the 10 % sodium dodecyl sulphate–polyacrylamide gel (10 % SDS-PAGE). Then samples were electrophoresed on this gel. Afterward, the protein was transferred to the polyvinylidene difluoride (PVDF) membrane (Beyotime, China). The membrane was then occluded with Tris-buffered saline (TBS) containing 0.1 % (v/v) Tween-20 and 5 % (w/v) skim milk, and incubated at room temperature for 1 h. Following this, the membrane was incubated for a duration of 2 h with the primary antibody dissolved in Primary Antibody Dilution Buffer (Beyotime, China) or blocking buffer. Primary antibodies used in this research were as follows: anti-PEDV N mouse mAb [7] provided by Shanghai Veterinary Research Institute, Chinese Academy of Agricultural Sciences, anti-Flag mouse mAb (Abmart, USA), anti-β-actin mouse mAb (Abmart, USA). Then wash the membrane five times with TBST, each time for 5min. After that, membranes were incubated in secondary antibody dilution solution containing Goat Anti-Mouse IgG-HRP for 45min. Finally, an ECL western blotting substrate was applied to the membranes and protein staining on PVDF membranes was analyzed using the Tanon-5200 multi-infrared imaging system for analysis.

### 2.5 Real-time RT-PCR

Total RNA was extracted from Vero cells using EZ-10 DNAaway RNA Mini-Preps Kit (Sangon Biotech, China) and reverse-transcribed using Hifair® Ⅱ 1st Strand cDNA Synthesis SuperMixaccording (YEASEN，China) in accordance with the manufacturer's protocol. Sequence of qRT-PCR primers used in this study were collected in Supplementary Table S1. The qRT-PCR was performed with Hieff® qPCR SYBR Green Master Mix (YEASEN, China) in a CFX96 Real-Time System (Bio-Rad). We used β-actin as internal reference control and adopted the 2^−ΔΔCt^ method to analyzed the real-time PCR results（Table S1 shows the sequences of primers used in PCR).

### 2.6 Tissue culture Infectious dose (TCID_50_) assays

The cells were inoculated one night in advance into 96-well plates. Use phosphate-buffered saline (PBS) to wash the plates after the cells growing to form a monolayer. Following this, the cells were infected with a PEDV virus solution that was serially diluted ten-fold, with eight dilution wells per dilution. The culture plates were then placed inside a CO_2_ incubator and observed every day for up to five days. The wells in which syncytium formation had occurred were labeled as "positive." Following the Reed-Muench methodology, the virus solution's titration was calculated.

### 2.7 Indirect Immunofluorescence assay (IFA)

At the beginning, well-growing cells were plated and then incubated with PEDV (multiplicity of infection [MOI] = 0.1) and added varying concentrations of remdesivir. After a three-time washing with PBS, 80 % cold ethanol was added to each well to completely immerse the cells. The cells were then fixed by incubating them at 4 °C for 1 h. Post fixation, the fixation solution was discarded, and the wells were washed three times. Subsequently, the cells were cultivated at 37 °C for 1 h in anti-PEDV N mouse mAb. Following that, the cells were washed thrice, and Goat Anti-Mouse IgG AF 488 (Abmart, USA) was added to each well. The samples were protected from light and stored at 37 °C for 45 min. After being washed thrice with PBS, we used the Invitrogen EVOS FL Auto Cell Imaging System to capture images.

### 2.8 Cytotoxicity assay

We purchased the Cell Counting Kit-8 (CCK8, TOPSCIENCE, China) to assess the cytotoxicity of remdesivir was assessed in 293T cells, Vero cells and LLC-PK1 cells, in accordance with the user guide. First, cells were grown in 96-well plates to 80 % confluence for 24h. After washing the plates, they were supplied with increasing concentration from 5 μM to 100 μM. Cells treated with DMSO were utilized as the control. CCK8 was added after 24h. Then the samples were stored at the carbon dioxide cell culture incubator for 1h. Absorbance at 450 nm was measured with the 722 N visible spectrophotometer (INESA, China).

### 2.9 Viral attachment, internalization replication and release assay

To evaluate virus attachment, Vero cells were infected with PEDV (MOI = 0.1) at 4 °C for 1h, under different concentrations of remdesivir or without it, which ensured virus binding but not internalization. After discarding the culture medium, use pre-cooled PBS to wash the cells thrice. Extract total RNA for Rt-PCR.

To evaluate virus internalization, Vero cells were incubated with PEDV (MOI = 0.1) at 4 °C for 1h. The cells were washed thrice to ensure removal of unbound viruses, followed by incubation with varying concentrations of remdesivir at 37 °C for 2 h. Collect the cells and extract total RNA for Rt-PCR.

To evaluate virus replication, Vero cells were incubated with PEDV (MOI = 0.1) for 1h at 37 °C to ensure that more virus adsorbed and penetrated. Cells were cultured with multiple concentrations of remdesivir at 37 °C until the appearance of the cytopathic effect (CPE) in the control well. We observed and photographed the characteristic morphological changes of Vero cells. At the same time, we collected cells and the supernatants to evaluate viral loads and titers by Rt-PCR and TCID_50_ assay, respectively.

To evaluate virus release, after PEDV infection of the Vero cells for 10h, the medium was replaced, washed plates three times and added the culture solution containing remdesivir or DMSO. After incubation for 2h at 37 °C. The mRNA copies of PEDV-N were detected by RT-qPCR, after collection of the cell supernatant.

### 2.10 Cell-based PEDV RdRp activity assay

293T cells were seeded overnight and then transfected with pCDNA4.0-nsp 7, pCDNA4.0-nsp 8, pCAGGS-nsp12 and PEDV-Gluc using the Lipo 8000™ Transfection Reagent (Beyotime, China). Cells were treated with Remdesivir or DMSO after transfection for 6h. Coelenterazine-h was dissolved in propylene glycol to a concentration of 5 mM as the stock solution. Before every test, the stock solution was diluted in PBS to a concentration of 50 μM and stored at room temperature for 30min. The entire process must be conducted in the absence of light. To perform the luminescence test, the well of a 96-well plate was added into 10 μL of supernatant from samples. Luminescence was measured for 0.5 s immediately after injecting 60 μL of working fluid using multifunctional microplate readers (BioTek Synergy2) (BioTek Synergy2, American).

### 2.11 RNA-seq analysis

At the beginning, quality-check FASTQ files produced from RNA-Seq by FastQC (v.0.11.8) (https://www.bioinformatics.babraham.ac.uk/projects/fastqc/). Then remove the low-quality and adapter sequences by Cutadapt (v1.15). The reference genome and gene model annotation files for Chlorocebus sabeus 1.1 were downloaded directly from the genome website (ftp://ftp.ncbi.nlm.nih.gov/genomes/all/GCF_000409795.2 Chlorocebus_sabeus_1.1). Subsequently, use HISAT2 (v2.0.5) to build the reference genome index and align the paired-end clean reads to the reference genome. HTSeq version 0.9.1 was utilized to quantify the number of reads that were mapped to each gene, and the resulting count of reads for each gene was used to determine its expression level. FPKM (Fragments Per Kilobase Million) is a widely used method for normalizing gene expression levels. It represents the number of reads per kilobase of length for a particular gene in every one million total reads. DESeq software (1.20.0) was used for differential expression analysis between two comparison groups. DESeq analysis was used to select differentially expressed genes with the conditions of an expression difference fold change |log2FoldChange|>1 and a significant P-value <0.05. GO enrichment analysis were produced from the topGO, and the P-value was calculated by the hypergeometric distribution method. We stipulated the threshold for significant enrichment was P-value<0.05. We used P-value to identify significant GO terms that were significantly enriched by differentially expressed genes to determine the main biological functions of differentially expressed genes. ClusterProfiler (3.4.4) software was used to perform the KEGG pathway enrichment analysis, focusing on significantly enriched pathways with a P-value <0.05.

### 2.12 Statistical

All date presented as the means ± SD from at least three independent experiments and were analyzed using GraphPad Prism 8.0.1 software. Difference between the two indicated settings were considered statistically significant if p < 0.05 (*), p < 0.01 (**), p < 0.001 (***), p < 0.0001 (****). N.s indicates non-significance.

## 3 Results

### 3.1 Antiviral effects of remdesivir on the PEDV

**Fig. 1:**
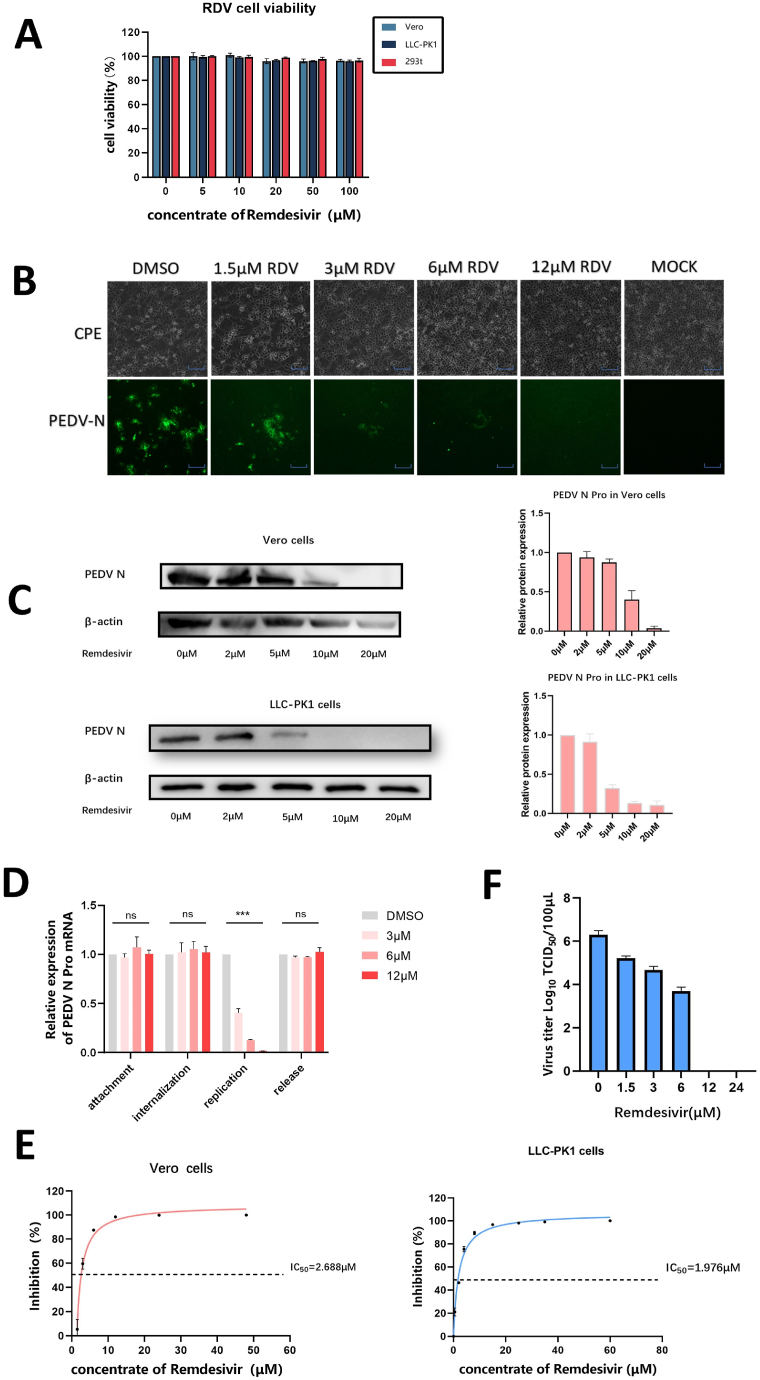
Effect of remdesivir on PEDV. (A) Cytotoxicity of remdesivir toward Vero cells, LLC-PK1 cells and 293T cells was analyzed by CCK8 assay. The relative viability of Vero cultured without drug was set to 100 %. Each value represents the average of three independent experiments. (B) Antiviral activity of remdesivir against PEDV was assessed through CPE observation and IFA. Scale bar: 100 μM. (C) The protein level of PEDV N and grayscale analysis in Vero cells and LLC-PK1 cells. (D) Effect of remdesivir on attachment, internalization replication and release of PEDV in Vero cells. (E) Dose-dependent curves of remdesivir against PEDV in Vero cells and LLC-PK1 cells. (F) Reduction of PEDV titers by remdesivir in Vero cells. Statistical significance is denoted by ***P < 0.001.

After evaluating the cytotoxicity of remdesivir in Vero cells (Fig. 1A), we assessed anti-PEDV effects of remdesivir. Cells infected with PEDV were incubated (MOI = 0.1) at 37 °C for 1 h and cultured until clear signs of CPE were observed in the control group. In the whole process, cells were immersed in medium containing different concentrations of remdesivir. As show in Fig. 1B，Vero cells infected with PEDV exhibited favorable conditions when treated with 12 μM, similar to that of the Mock group. As the drug concentration was lowered, an increasing amount of CPE was observed in the wells. The IFA indicated that the density of green fluorescence increased as drug concentration decreased (Fig. 1B). The Western blot analysis showed a dose-dependent reduction in expression of PEDV N protein, with significantly elimination levels at 20 μM. In LLC-PK1 cells, PEDV N protein expression can be completely inhibited by 10 μM of remdesivir (Fig. 1C).

The life cycle of coronaviruses typically consists of four distinct phases: attachment, internalization, replication, and release. In this study, we assessed the impact of different concentrations of remdesivir on the life cycle of PEDV in Vero cells. Our results indicate that remdesivir only inhibits PEDV replication (Fig. 1D). We plotted the inhibition curve of remdesivir and found that the IC_50_ was 2.668 μM in Vero cells and 1.976 μM in LLC-PK1 cells (Fig. 1E). At the same time, we measured the virus titers by collecting the Vero cells’ supernatant and found that the virus titers got lower markedly as the concentrations of drug increased (Fig. 1F). These results indicate that Remdesivir inhibits PEDV during replication.

### 3.2 Remdesivir inhibits PEDV RdRp activity

**Fig. 2:**
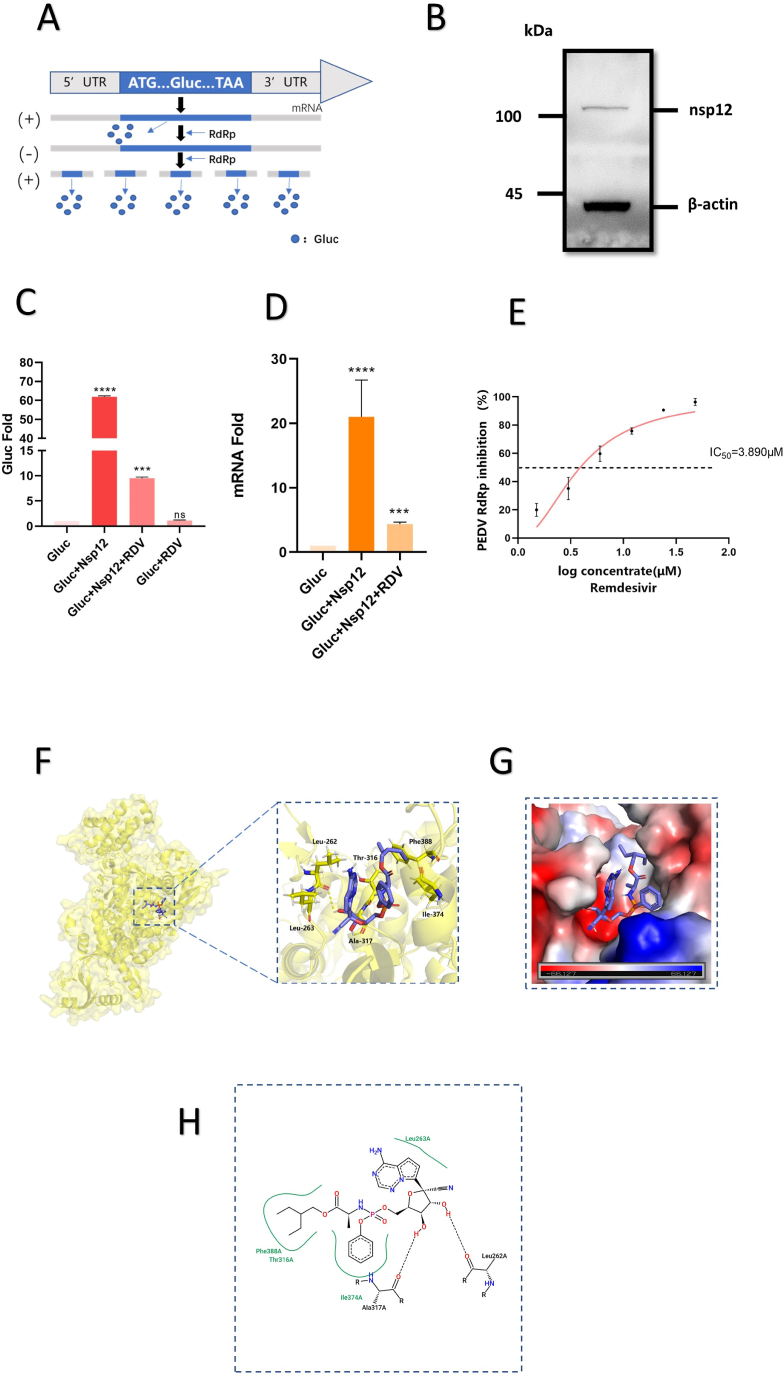
Remdesivir inhibits PEDV RdRp activity. (A) Schematic diagram of PEDV-RdRp-Gluc reporter system. (B) Expression of the PEDV Nsp12. (C) Gluc plasmid and pCAGGS-Nsp12 were transiently transfected into 293T cells. After 6h, cells were treated with 12 μM remdesivir for 18h. The cell supernatant was taken to measure the Gluc activity. (D) Levels of Gluc mRNA expression were showed by RT-qPCR. (E) The IC_50_ for remdesivir was determined using non-linear regression analysis. (F) Proposed binding mode of RMP against PEDV Nsp12. (G) Diagram of binding pocket of PEDV Nsp12 with RMP. (H) Interaction between the PEDV Nsp12 and RMP by PoseView. The corresponding diagram produced by the website version of PoseView (https://proteins.plus/). All date, Mean ± SEM values are shown, based on data from three independent experiments that are representative. RMP, remdesivir monophosphate. Difference between the two indicated settings were considered statistically significant if p < 0.001 (***), p < 0.0001 (****).

As a type of nucleoside analog, remdesivir typically targets the virus RdRp. To investigate the interaction between remdesivir and PEDV RdRp, we employed a PEDV RdRp activity reporter assay system referred to Zhao's work [6]. As demonstrated by Fig. 2A, the cytomegalovirus (CMV) promoter initiates the transcription of Gluc. Subsequently, the expression of PEDV RdRp leads to an increase in the amount of Gluc RNA, resulting in elevated levels of Gluc mRNA and protein. Therefore, the luminescence test measuring Gluc activity reflects the activity of PEDV RdRp. First, we confirmed the expression of PEDV nsp12 in 293T cells (Fig. 2B). We transfected PEDV-Gluc reporter-expressing cells with a nsp12-expressing vector, which resulted in a 60-fold increase in fluorescent value as compared to cells expressing only the reporter plasmid (Fig. 2C). The results of RT-qPCR were similar to the variation of Gluc activity fold (Fig. 2D). Based on the aforementioned data, we confirmed that PEDV nsp12 is capable of enhancing Gluc activity.

Next, we evaluated the direct inhibitory effects of remdesivir on PEDV RdRp activity. Concentrations of up to 100 μM did not exhibit any cytotoxicity in 293T cells (Fig. 1B). Pre-experiments confirmed that there was no interference with background values in the presence of the drug (Fig. 2D). At 6 h post transfection, cells co-transfected with Gluc and nsp12 plasmids were treated with multiple concentrations of remdesivir. The results showed that remdesivir dose-dependently inhibited Gluc activity (Fig. 2E), indicating the drug's ability to inhibit PEDV RdRp.

To obtain a thorough comprehension of remdesivir's impact on RdRp, we performed molecular docking. We first utilized the AlphaFold2 web tool to predict the structure of PEDV nsp12 protein and then exploited AutoDock Vina v.1.2.0 software to determine the docking site between remdesivir monophosphate (RMP) and PEDV nsp12 (Fig. 2F and G). Based on this, we displayed a 2D docking result image by the website https://proteins.plus/, which indicates the interaction markers between the ligand and the receptor (Fig. 2H). Our docking results revealed the formation of hydrogen bonds between RMP and Leu 262 and Ala 317, in addition to hydrophobic interactions with 263 Leu, 316Thr, 374 Ile, and 388 Phe. These findings offer novel insight regarding remdesivir's mechanism of inhibiting PEDV RdRp.

### 3.3 Effect of remdesivir on inflammatory factors

**Fig. 3:**
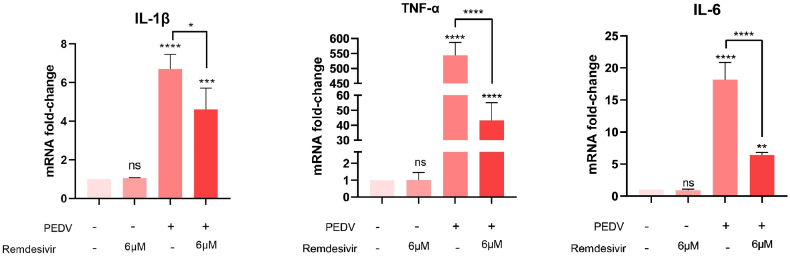
Effects of remdesivir and PEDV on the expression of IL-1β (A), TNF-α (B), and IL-6 (C). Difference between the two indicated settings were considered statistically significant if p < 0.001 (***), p < 0.0001 (****). ns indicates non-significance.

IL-1β, TNF-α and IL-6 have been reported as crucial inflammatory factors during PEDV infection [ [8,9]]. To extensively explore remdesivir's anti-PEDV mechanism, we examined its effects on the levels of essential inflammatory factors. The result is as shown in Fig. 3. There were the same changes of mRNA amount among three inflammatory factors. In Vero cells treated with 6 μM remdesivir, the levels of inflammatory factors remained unaltered. PEDV induced the transcription of these cytokines, particularly TNF-α, whose quantities increased by approximately 550-fold. Furthermore, remdesivir reduced the transcription levels of these factors. Viral infection triggers an extensive cytokine response, such as IL-1, IL-2, IL-6, TNF-α, IFN-γ, IFN-induced protein 10 kDa and so on. It results in an "inflammatory storm" that leads to significant cell damage. The “inflammatory storm” induced by SARS-CoV-2 infection can result in severe damage to immune cells through a process known as apoptosis. Additionally, the excessive accumulation of immune cells and fluid in the lungs can obstruct the airflow, potentially resulting in fatality [10]. Our findings indicated that remdesivir alleviates inflammatory damage by inhibiting the virus instead of targeting the production pathways of inflammatory factors.

### 3.4 Exogenous Nucleosides Attenuate Antiviral Effect of Remdesivir

**Fig. 4:**
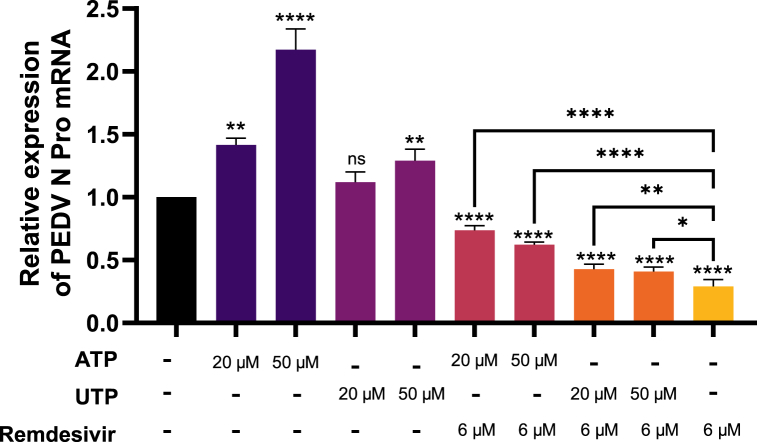
Exogenous nucleosides promote viral replication and attenuate antiviral effect of remdesivir. PEDV-infected Vero cells (MOI = 0.1) were incubated in medium containing remdesivir, ATP or UTP at different concentrations, and PEDV N Pro mRNA level were obtained by RT-qPCR. Difference between the two indicated settings were considered statistically significant if p < 0.05 (*), p < 0.01 (**), p < 0.001 (***), p < 0.0001 (****).

Given that remdesivir is a nucleoside analogue, we added exogenous nucleosides to study their impact on the drug's efficacy. Overall, the replication of PEDV was enhanced by ATP and UTP, while the antiviral potency of remdesivir was attenuated. Actually, 20, 50 μM ATP and 50 μM UTP significantly increased PEDV mRNA synthesis, but not 20 μM UTP. ATP and UTP promoted the replication of PEDV and reduced remdesivir's antiviral effect. Specifically, 20, and 50 μM ATP, and 50 μM UTP significantly increased PEDV mRNA synthesis, whereas 20 μM UTP did not. Furthermore, the presence of exogenous nucleosides considerably reduced PEDV inhibition (Fig. 4). These findings imply antagonism between remdesivir and natural nucleosides.

### 3.5 RNA-seq analysis after treatment

**Fig. 5:**
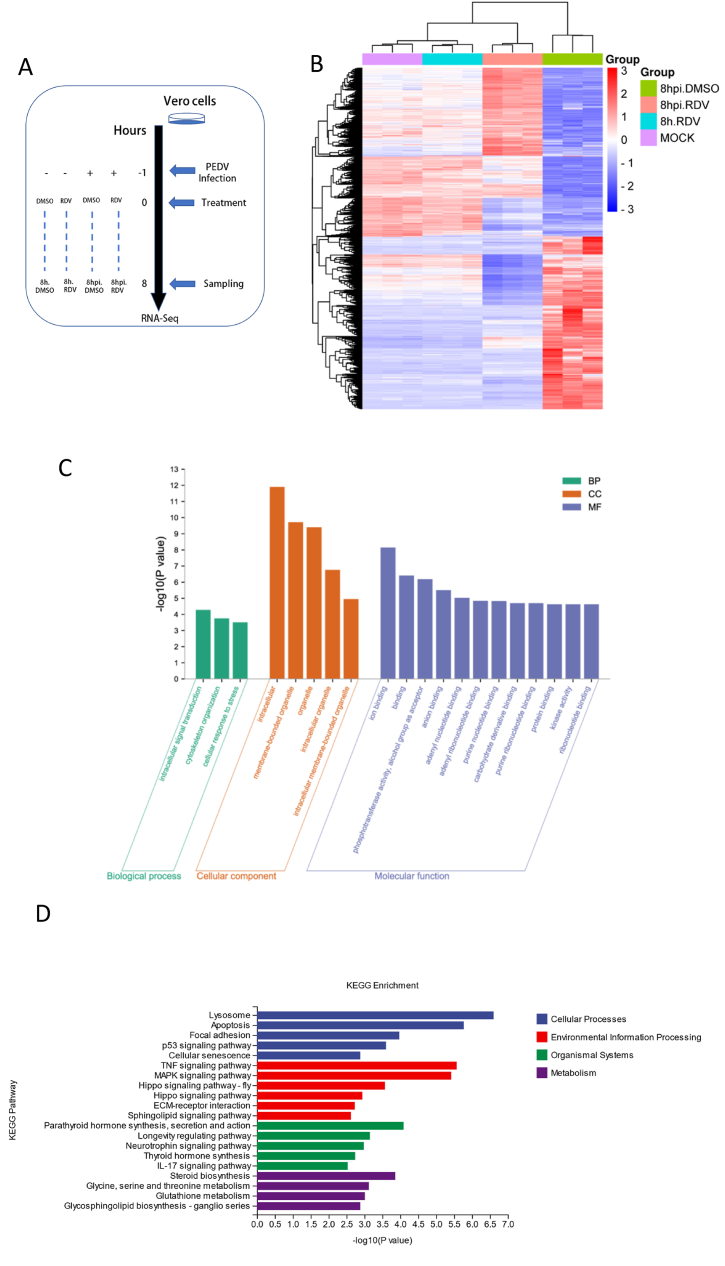

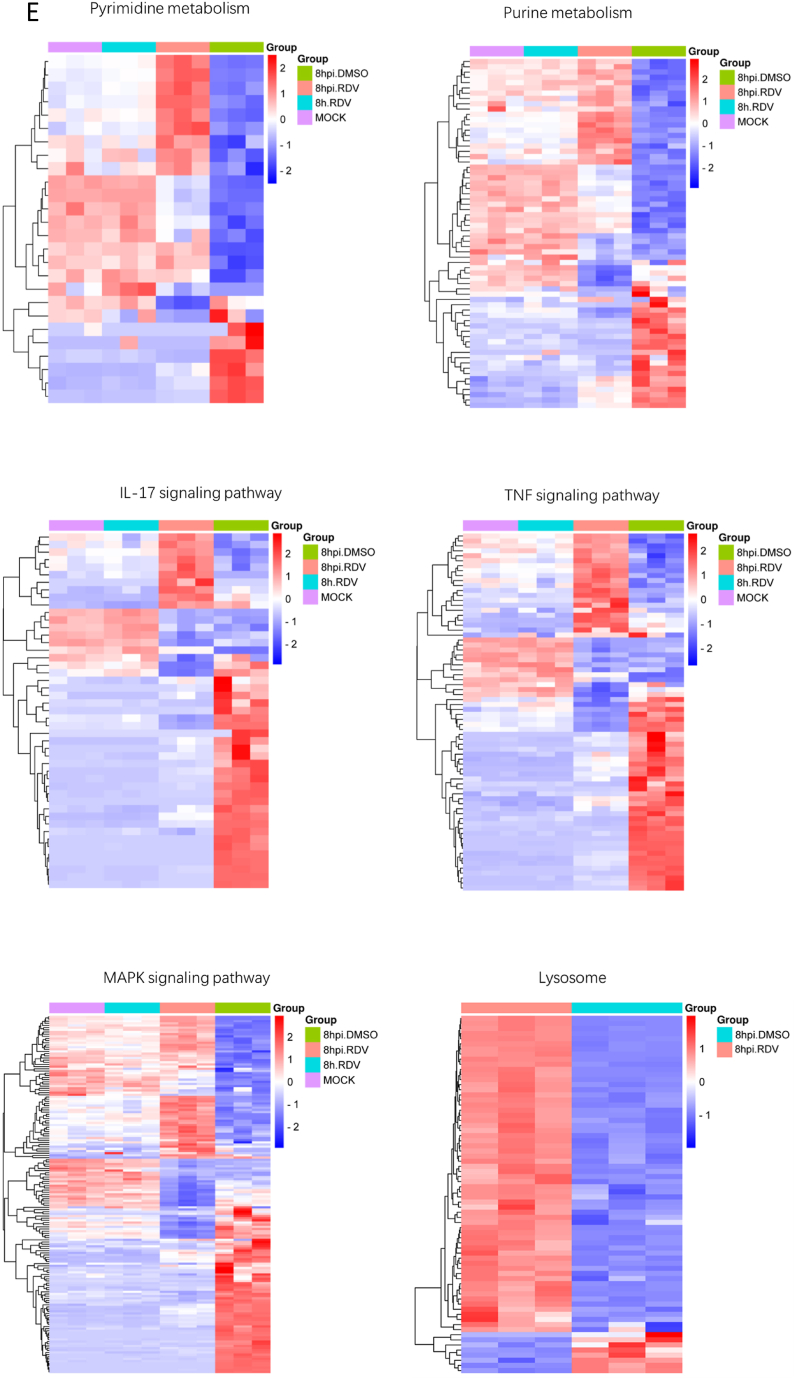
Transcriptional analysis of remdesivir treatment. (A) Timeline of the transcriptomic experiment (MOI = 0.2). RDV, remdesivir. (B) Heatmap of differential gene (Differential expression fold | log2FoldChange | > 1, significant P-value <0.05) clustering analysis across all samples. Genes were clustered using the complete method. (C) The top 20 GO enrichments from group “8hpi.DMSO vs 8hpi.RDV”. BP, biological process. CC, cellular component. MF, molecular function. (D) The top 20 KEGG enrichments from groups “8hpi.DMSO vs 8hpi.RDV”. (E) Heat map of the genes enriched in Purine metabolism, Pyrimidine metabolism, IL-17 signaling pathway, TNF signaling pathway, MAPK signaling and lysosome. “8hpi.DMSO”: the cells infected with PEDV for 8 h under DMSO treatment; “8hpi.RDV”: the cells infected with PEDV for 8 h under 8 μM remdesivir treatment; “8h.RDV”: the normal cells for 8 h under 8 μM remdesivir treatment; “MOCK”: the normal cells for 8 h under DMSO treatment.

RNA-seq was used to investigate the antiviral effects of remdesivir and understand its inhibition mechanism. The Genome Sequence Archive (GSA: **CRA011154**), which contains the raw sequence data mentioned in this paper, has been deposited at the National Genomics Data Center in China. Vero cells infected with or without PEDV and treated with remdesivir or DMSO for 8 h were used for transcriptional analysis (Fig. 5A). The cluster analysis of differentially expressed genes showed that remdesivir treatment reversed the changes induced by the PEDV infection, and shifted the transcriptome towards the mock group (Fig. 5B). GO analysis results indicated that most of the top 20 significant enriched GO terms belonged to molecular function (MF) category, with terms related to nucleotide binding being the most significant (Fig. 5C). This suggested that remdesivir might play a role in nucleotide-related biological processes. Analysis of the heat map of genes involved in nucleotide metabolism indicated that remdesivir inhibited the hijacking of PEDV to Vero cells (Fig. 5E). Pathway analysis based on the KEGG database identified the top 20 significant signaling pathways (Fig. 5D). Enrichment analysis revealed many immune inflammation-related pathways including the IL-17, TNF, and MAPK signaling pathways. Heat maps based on these pathways showed that remdesivir treatment could often reverse the effects of PEDV infection (Fig. 5E and Table S2). The lysosomal pathway, which has antiviral properties, was found to be the most significant pathway. The heat map of this pathway showed that almost all genes were up-regulated in response to remdesivir treatment, therefore reflecting an enhanced lysosomal-mediated host defense mechanism (Fig. 5E).

## 4 Discussion

PEDV infection is highly fatal for newborn piglets, posing a significant challenge to the global swine industry's growth. Attenuated and inactivated vaccines are the prevailing methods for preventing PEDV infection. However, the emergence of several variant PEDV strains globally has led to a reduction in the vaccine's protective efficacy, thereby complicating efforts in epidemic prevention and control. Hence, there is an urgent need to develop novel therapeutic drugs to combat the PEDV virus effectively. Some promising drugs have been previously reported. For instance, Buddlejasaponin IVb, a major component of Pleurospermum kamtschaticum, has the potential to inhibit PEDV *in vitro* and *in vivo* by reducing the activation of NF-κB signaling pathway [11]. Additionally, cinchonine has shown to inhibit PEDV infection by inducing autophagy [12].

RdRp plays a crucial role during the life cycle of RNA viruses, thereby making it a promising target for antiviral drugs. Antiviral drugs targeting RdRp, including nucleoside analogues such as favipiravir, ribavirin, and galidesivir, have a vital role in combating human viruses [ [[13], [14], [15]]]. Remdesivir, a nucleoside analogue, became the first COVID-19 treatment approved by the FDA [5]. In this study, we confirmed the significant anti-PEDV activity of remdesivir.

We conducted research on Vero cells and LLC-PK1 cells using the PEDV Shpd2012 strain which belongs to PEDV genotype 2a (G2a) [16]. The findings revealed that remdesivir inhibited PEDV replication with an IC_50_ of 2.668 μM in Vero cells and IC_50_ of 1.976 μM in LLC-PK1 cells. Remdesivir targets RdRp, which is a well-known fact. Using cryogenic electron microscopy (cryo-EM), structural basis for inhibition of SARS-CoV-2 RdRp by remdesivir have been reported [4]. At the same time, the cell-based assays targeting MERS-CoV and SARS-CoV-2 RdRp were established successively [ [6,17]]. In these assays, remdesivir showed strong inhibitory effects. We also developed a PEDV RdRp activity reporter system to evaluate the effectiveness of remdesivir. The assay demonstrated that the inhibition effect of remdesivir on RdRp was dose-dependent. We performed molecular docking simulations using Autodock Vina and found that Remdesivir can form hydrogen bonds and hydrophobic interactions with Nsp12, which may be the mechanism behind the inhibition of RdRp activity. Nonetheless, more research is warranted to obtain an in-depth understanding. As is well known, SARS-CoV-2 infection induces cytokine storm, causing rapid disease progression and high mortality rates [18]. To examine the effect of remdesivir on cytokines, we selected three representative inflammatory factors associated with PEDV infection [ [8,9]]. The results demonstrated that remdesivir reduced inflammatory damage by targeting the virus and not the production pathways of inflammatory factors. Remdesivir is a phosphoramidate prodrug that undergoes cellular metabolism to yield an active NTP analog called remdesivir triphosphate (RTP) [19]. Enzyme kinetics revealed that Ebola virus (EBOV) RdRp and respiratory syncytial virus (RSV) RdRp incorporate ATP and RTP with similar efficiencies [20]. Exogenous nucleosides were added to Vero cells treated with remdesivir, which slightly weakened the drug's antiviral effectiveness. These findings suggest an antagonistic relationship between remdesivir and exogenous nucleosides. In line with this, low doses of ATP have been shown to promote replication of African swine fever virus (ASFV) and reduce the inhibitory effect of GS-441524, the bioactive compound in remdesivir, on ASFV [21]. RNA-Seq is frequently applied to investigate the virus-host interaction. In a former study, RNA-Seq was employed to uncover that swine acute diarrhea syndrome coronavirus (SADS-CoV) infection noticeably perturbed the gene expression patterns of the host cell [22]. In addition, Single cell RNA sequencing (scRNA-Seq) have yielded valuable insights into the pathogenesis of SARS-CoV-2 [23]. In this study, we utilized RNA-Seq to examine the influence of remdesivir on PEDV-infected Vero cells. While treatment with remdesivir showed minimal effect on the MOCK group, it caused an overall shift in the transcriptome profile towards that of the MOCK group. It was observed that a significant proportion of genes expressed differentially in the lysosome pathway were up-regulated following remdesivir treatment. The autophagy-lysosome pathway plays a crucial role in innate immunity as it serves as the primary defense mechanism against pathogen elimination. Recent research has uncovered that coronaviruses (CoVs) have evolved diverse strategies to evade detection by the autophagy-lysosome pathway. It has been discovered that SARS-CoV-2 ORF3a actively suppresses autophagic flux by impeding the fusion of autophagosomes with lysosomes [24]. Furthermore, both SARS-CoV-2 and MERS have the ability to suppress the activity of Rab 7, impeding the fusion and acidification process between lysosomes and autophagosomes [25]. Finally, studies have identified that ORF5 and ORF4b of MERS-CoV downregulate the expression of genes associated with the lysosomal membrane and lysosomal acidification [26]. Further investigations are essential to clarify the interaction between remdesivir and the host's lysosomal pathway.

Similar to remdesivir, molnupiravir, a nucleoside analogue, exhibits potent inhibition against coronaviruses, making it the world's inaugural oral medication for COVID-19 treatment. According to previous research in our laboratory, molnupiravir also exhibited significant inhibition effects against PEDV, with an IC50 of 12.30 μM [27]. In comparison to molnupiravir, remdesivir demonstrates a stronger inhibitory effect on PEDV, possibly due to the contrasting inhibition mechanisms of the two drugs. Both drugs compete with natural nucleoside triphosphates (NTP) as substrates, with remdesivir competing with adenosine and molnupiravir competing with uracil. As previously mentioned, remdesivir halts the RNA chain and induces chain termination, whereas molnupiravir, upon incorporation into the chain, triggers numerous genetic mutations [28]. These mechanisms prove fatal to coronaviruses, with the chain termination mechanism being particularly effective in virus eradication.

## 5 Conclusions

Here, we show that remdesivir significantly inhibit the replication of PEDV in Vero cells and LLC-PK1 cells. Remdesivir targeting PEDV's RdRp demonstrated antiviral effects during treatment. The transcriptome changes of PEDV-infected cells were shift towards the mock -treatment control group under remdesivir treatment.

## Funding

This research was kindly supported by a grant from the National Key 10.13039/100006190Research and Development Plans of China (No. 2021YFD1800401) to Zhe Wang, 10.13039/501100001809National Natural Science Foundation of China (No. 32070128) to Zhe Wang, Shanghai Biomedical Science and Technology Support Special Project (No. 21S11900200) to Zhe Wang, 10.13039/501100001809National Natural Science Foundation of China (No. 31472211) to Zhi-Biao Yang, and Natural Science Foundation of Shanghai (No. 21ZR1433900) to Zhi-Biao Yang.

## Data availability statement

The Genome Sequence Archive (GSA: CRA011154), which contains the raw sequence data mentioned in this paper, has been deposited at the National Genomics Data Center in China. The data will be available on 2024-05-01.

## CRediT authorship contribution statement

**Zi-Xin Huang:** Data curation, Formal analysis, Investigation, Methodology, Validation, Visualization, Writing – original draft, Writing – review & editing. **Shu-Ting Zhou:** Investigation, Methodology. **Jing Wang:** Methodology, Resources. **Zhi-Biao Yang:** Conceptualization, Funding acquisition, Investigation, Methodology, Resources. **Zhe Wang:** Conceptualization, Funding acquisition, Investigation, Project administration, Resources, Supervision, Writing – original draft, Writing – review & editing.

## Declaration of competing interest

The authors declare that they have no known competing financial interests or personal relationships that could have appeared to influence the work reported in this paper.
